# Inhibition of ADAM-17 more effectively down-regulates the Notch pathway than that of γ-secretase in renal carcinoma

**DOI:** 10.1186/1756-9966-32-26

**Published:** 2013-05-09

**Authors:** Zhen Guo, Xunbo Jin, Haiyan Jia

**Affiliations:** 1 Minimally Invasive Urology Center, Provincial Hospital Affiliated to Shandong University, No. 324 Jingwu Road, Jinan 250001, China; 2 Shandong traffical hospital, No.11 Wuyingshan Road, Jinan, 250000, China

**Keywords:** ADAM-17, Notch, Renal cell cancer, Apoptosis, Marimastat, γ-secretase

## Abstract

**Background:**

Our study is to research the effect of inhibited ADAM-17 expression through the Notch pathway in renal carcinoma.

**Methods:**

Immunohistochemistry and western blot were used to examine the expression of ADAM-17 protein in renal cancer tissues. Proliferation and cell invasion of 786-o cells, as well as OS-RC-2 cells, after treatment with two different inhibitors of the Notch pathway, were examined by CCK-8 assay and Transwell assay, respectively. 786-o cell apoptosis was measured using the FCM test.

**Results:**

ADAM-17 was highly expressed in RCC tissues. Compared with blocking γ-secretase, a known mechanism of impairing Notch, blockade of ADAM-17 more effectively down-regulated the expressions of Notch1 and HES-1 proteins. Similarly, we found that the ADAM-17 inhibitor, Marimastat, could more efficiently reduce renal cell proliferation and invasive capacity in comparison with the γ-secretase inhibitor DAPT when used at the same dose. Similar results were obtained when apoptosis of 786-o was measured.

**Conclusion:**

Compared with γ-secretase, inhibition of ADAM-17 expression more effectively inhibits Notch pathway-mediated renal cancer cell proliferation and invasion. ADAM-17 may be a new target for future treatment of renal carcinoma.

## Background

The molecular mechanisms underlying renal carcinoma (RCC) are still unclear. Moreover, because RCC easily metastasizes, despite conventional treatments the prognosis remains poor. Apoptosis and cell differentiation of RCC is believed to be controlled by multiple cell pathways. Thus, much research is focused on developing targeted therapies at the molecular level of RCC.

Current research of the Notch signaling system is mostly focused on the pathway and its corresponding target genes, while little research is centered on activation of the Notch pathway. To this end, it is known that the Notch signaling pathway is activated by a 3-step proteolysis process involving three proteolytic cleavage sites known as S1, S2 and S3 [1-3]. Proteolysis on the S2 site, which is critically affected by the key enzyme ADAM-17 (also called TACE), is especially overlooked. The ADAM-17 gene is located on human chromosome 2 (2p25) and rat chromosome 12. It is 50 kb in length and composed of 19 exons. It has a similar structure to most ADAMs with a front control region, metalloproteinase peptidase region, integrin-splitting region, cysteine-rich region, transmembrane region and intracellular region [4,5]. ADAM-17 plays a crucial role in the development of epithelial tumors. High expression of ADAM-17 may further increase release of epidermal growth factor receptor (EGFR) ligands including EGF, androgen receptor (AR), heparin-binding (HB)-EGF, transforming growth factor (TGF-α) and epiregulin (EPR), that result in the over-activation of EGFR which, in turn, plays a significant role in cleaving the S2 site in the Notch signal pathway.

The enzyme γ-secretase has also been found to trigger activation of the Notch pathway by splitting the S3 site. According to the research of Zhu [6], blockade of γ-secretase inhibits activation of the Notch pathway. In contrast to ADAM-17, which cleaves the S2 site in the extracellular region, γ-secretase acts on a transmembrane region. Thus we hypothesized that because of increased accessibility to the extracellular region the inhibition of ADAM-17 could more significantly down-regulate Notch activation, than that of γ-secretase. Testing of this hypothesis confirmed that ADAM-17 is a key enzyme for the activation of the Notch signal pathway. Moreover, inhibition of its activity more effectively promotes apoptosis and impairs invasive ability in RCC than that of γ-secretase with DAPT. Therefore, the ADAM-17 inhibitor Marimastat is a better targeted inhibitor of the Notch pathway than the γ-secretase inhibitor, DAPT.

## Materials and methods

### Collection of primary clear cell renal carcinomas

Sixty-seven pairs of clear cell renal carcinoma (CCRCC) tissues and 10 adjacent normal kidney tissues were collected at the Department of Urology of the Shandong Provincial Hospital of China. All RCC cases were confirmed clinically and pathologically to be of the clear cell type. All tumor specimens were staged based on the 2002 AJCC TNM classification of malignant tumors (Table 1). The samples were snap-frozen in liquid nitrogen and stored at -80°C until analysis. Prior written informed consent was obtained from all patients and the study was approved by the Protection of Human Subjects Committee of the hospital.

**Table 1:** Expression of ADAM-17 in renal carcinoma tissues

**Pathological factors**	**n**	**ADAM-17 positive**	**ADAM-17 negative**	***χ***^**2**^	**P**
TNM stage				16.39	<0.01
I	14	3	11		
II	22	14	8		
III	25	21	4		
IV	6	5	1		
Rate		64.18%	35.82%		

64.18% of positive expression of ADAM-17 was recorded in all 67 cases of renal carcinoma tissues, there are 26 positive cases in stage-III and stage-IV renal carcinoma and 5 negative cases, which indicates that ADAM-17 expression is more in high stages of RCC; despite the low expression rate in stage-I renal carcinoma, the ADAM-17 expression is increased as the tumor stage increasing(*χ*^2^=16.39, P<0.01).

### Immunostaining

Formalin-fixed, paraffin-embedded tissue sections were dewaxed in xylene, rehydrated in graded alcohols, and briefly microwaved in 0.001 mol/L citrate buffer (pH 6), to optimize antigen retrieval. Sections were then used to detect ADAM-17 using the Histostain-plus kit (BD Science, NY, US) according to the manufacturer’s instructions. The primary antibody of activated ADAM-17 (Abcam Ltd. Cambridge, UK) was diluted 1:500. Immunostaining was visualized using a Nikon microscope. The criteria of ADAM-17 positive expression are the more than 3 cells can be stained to the brown color at least three randomly selected 20xfields, however the negative is no staining.

### Cell culture and reagents

The CCRCC cell lines 786-O and OS-RC-2 were preserved in our laboratory. The cells were cultivated in RPMI 1640 medium and Dulbecco’s modified Eagle’s medium (Aidlab Biotechnologies Co. Beijing, China), respectively, and supplemented with 10% fetal calf serum in a humidified incubator at 37°C with a mixture of 95% air and 5% CO_2_.

### Cell treatment and grouping

We treated the 786-O and OS-RC-2 cells with ADAM-17 inhibitor, Marimastat (Tocris, UK) at concentrations of 1 μmol/L, 2 μmol/L and 3 μmol/L diluted in 1640 medium to a final volume of 2 ml, or the same concentrations of the γ-secretase inhibitor, DAPT (EMD bioscience, CA) for 24 hours. The control group was provided by cells incubated with 2 ml of 1640 medium alone. Afterwards cells were collected for further testing.

### Western blot

786-O cells and OS-RC-2 cells were lysed in radio-immunoprecipitation assay buffer and equal amounts of the protein extracts (30 μg per lane) were separated by 10% sodium dodecyl sulfate-polyacrylamide gel electrophoresis. Proteins were then transferred onto polyvinylidene fluoride membranes (Millipore, Billerica, MA) for western blotting. The primary antibodies against NOTCH1 (activated Notch intracellular domain), HES-1 (Abcam, Cambridge, MA), and β-actin (Aidlab Biotechnologies Co., Beijing, China) were incubated with membranes overnight at 4°C. After 3 washes, for 15 min each, in Tris-buffered saline supplemented with 0.1% Tween 20, membranes were incubated with peroxidase-conjugated goat anti-mouse/rabbit IgG antibodies (Aidlab Biotechnologies Co. Beijing, China) for 1 h at room temperature. The bound anti-bodies were visualized by an enhanced chemiluminescence detection system using medical X-ray films.

### Comparative inhibition of proliferation analysis with CCK-8 assay

Cells were seeded in a 96-well plate at approximately 8×10^4^ in a volume of 100 μl/well. Wells were also prepared that contained known numbers of four kinds of cells to be used to create a calibration curve. To measure apoptosis, 10 μl of the CCK-8 solution (Dojindo, Japan) was carefully added to each well of the plate. The plate was incubated for 1–4 h in the incubator during which time the absorbance was measured at 450 nm using a microplate reader at 30, 60, and 90 min.

### Transwell assay for cell invasion

Cell invasive ability was determined using the Transwell test kit (Corning, NY, USA). Briefly, matrigel was mixed with 1640 medium at a ratio of 1:7 and 100 μl was added to each upper-transwell then placed into the incubator for 1 hour for the mixture to set. Then, 786-O cells were serum-starved for 12 h in pre-warmed 1640 media alone to eliminate the effects of serum. Twenty-four hours after the application of matrigel, 600 μl of 10% FBS solution was added to the lower transwell. The serum starved cells were resuspended to a density of 2.5×105 in 1640 solution without FBS in a final volume of 1 ml, with or without Marimastat or DAPT. From this, 100 μl was added to each transwell (2.5×104). After 48 h in the incubator, the transwell casters were purged into PBS to remove the non-adherent cells, and then submerged it in 4% paraformaldehyde for 10 min for fixation, and finally replaced in PBS. After the membrane was dried, cells were observed and counted under a microscope (400×).

### Flow cytometry and cell cycle apoptosis

1×10^6^ cells were plated in 100 ml culture flasks and allowed to proliferate until 70–80% confluence was attained. Cells were then treated with Marimastat (1 μmol/L or 3 μmol/L), DAPT (1 μmol/L or 3 μmol/L), or DMSO (15 μl) as control. After 24 h, cells were washed then resuspended in PBS. To measure apoptosis, the Annexin-FITC Apoptosis Detection Kit (KAIJI BIOTECH, Nan Jing, CN) was used according to its instructions. Briefly, fresh cells were labeled with 1:500 diluted Annexin V-biotin conjugated with FITC followed by incubation with 1:1000 diluted PI. Annexin V-PI expression levels were measured by FACS Calibur (BD Science, NY, USA) and analyzed by Modfit Software.

### Statistical analysis

All data were analyzed using the SPSS statistical software package (SPSS Inc., Chicago, IL) All data were expressed as mean ± standard deviation (SD) unless otherwise specified. Intergroup differences for two variables were assessed by unpaired *t*-test. Differences in parameters between groups were evaluated by ANOVA followed by unpaired *t* test with Bonferroni correction for multiple comparisons. P<0.05 was considered statistically significant.

## Results

### ADAM-17 is over expressed in renal carcinoma tissues

**Figure 1:**
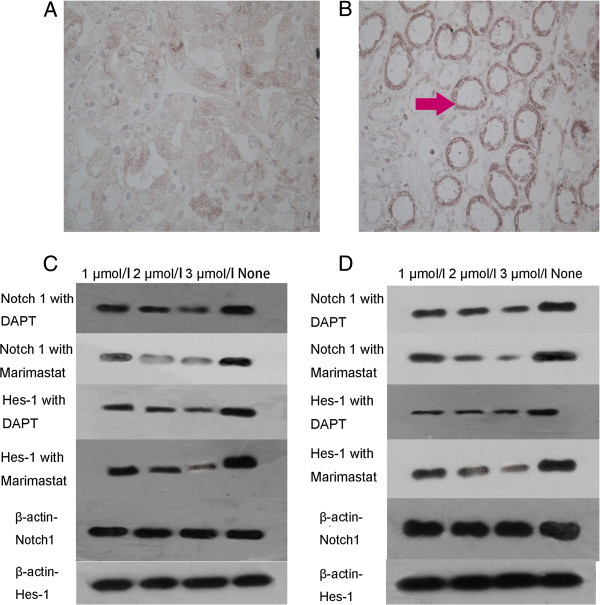
**Immumohistochemical staining of ADAM-17 in renal carcinoma tissues. A**: Normal kidney tissue stained by ADAM-17. **B**: Renal carcinoma tissue (stage-III) with ADAM-17 concentrated around the cytomembrane stained red (arrowed). **C**: Expression of Notch1 and HES-1 protein as measured by Western blot analysis after treatment with Marimastat or DAPT, or a media alone control, in 786-O cells. **D**: Expression of Notch1 and HES-1 protein levels by Western blot after treatment with Marimastat or DAPT, or a media alone control, in OS-RC-2 cells.

Through immunohistochemical staining assay we found that ADAM-17 was highly expressed in renal carcinoma tissues. Specifically, we observed 43 positive cases among a total of 67 cases (64.18%) (Figure 1A and B). The expression rate in the T1–T4 stages were 21.43%, 63.67%, 84.00% and 83.33%, respectively. ADAM-17 was highly expressed as the tumor stage increased, in the stageI, only 3/14 tissues were ADAM-17 positive but in the stage III and IV, the ADAM-17 positive tissue were increased to 21/25 and 5/6. To evaluate these results, we found that the positive expression rate of ADAM-17 was greater in the high tumor stage than low tumor stage (×2 = 16.39 P<0.01) (Table 1). In contrast, it was hardly expressed in non-renal carcinoma tissues. Indeed, from a total of 67 samples, only one sample was positive, resulting in a positive expression rate of 1.49% (P<0.05 data was not shown).

### Effects of the ADAM-17 inhibitor Marimastat and the γ-Secretase inhibitor DAPT on protein expression of Notch 1 and HES-1

**Figure 2:**
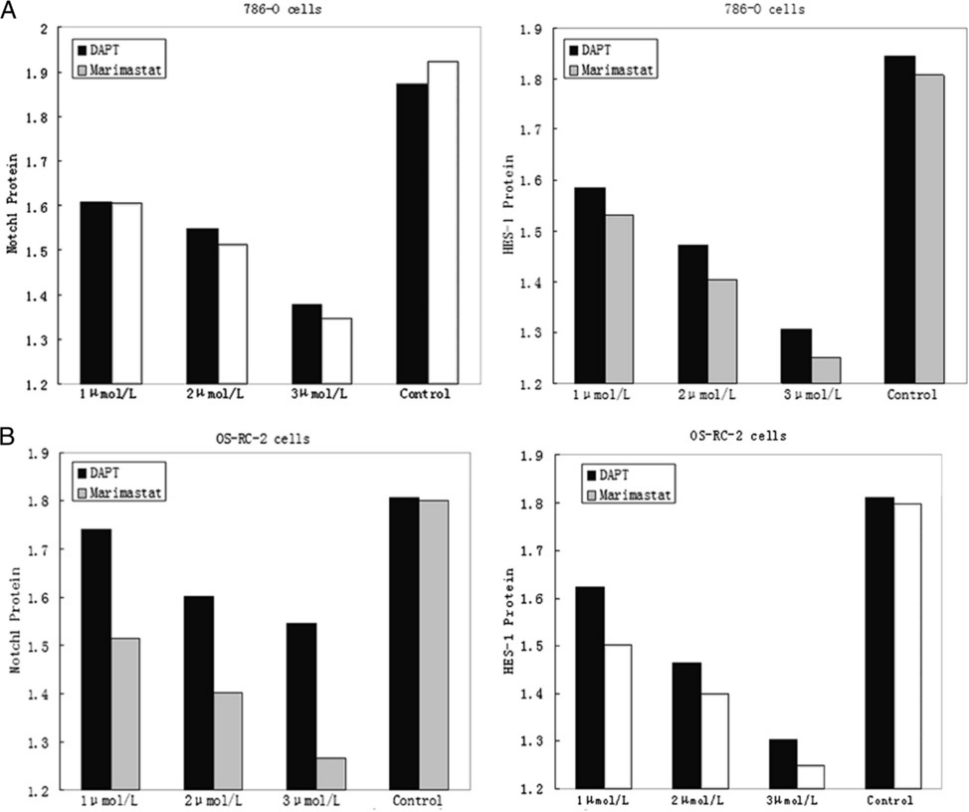
**Expression of Notch1 and HES-1 proteins in 786-O and OS-RC-2 cells. A**: Expression of Notch1 and HES-1in 786-O cells after treatment with Marimastat, DAPT, or control. **B**: OS-RC-2 cells were treated and analyzed as in ‘A.’

After treatment with either Marimastat or DAPT, the expression of Notch 1 and HES-1 proteins in 786-O and OS-RC-2 cells was examined by western blot. The Notch1 and Hes-1 protein level was measured by the concentration of the test group subtracted from the control group. We found that regardless of whether cells were treated by Marimastat or DAPT, expression of Notch 1 and HES-1 proteins was considerably decreased (P<0.05) (Figure 1C and D). The protein level of Notch1 and Hes-1 treated by Marimastat or DAPT were shown by (Figure 2A and B). Indeed, in 786-O cells, Notch 1 and HES-1 protein levels in 768-O cells treated by Marimastat decreased 0.397±0.126 and 0.411±0.096, respectively, while DAPT-treatment produced 0.364±0.068 and 0.391±0.099 decreases in Notch 1 and HES-1, respectively. Similar results were found in the OS-RC-2 cells, where Marimastat treatment decreased protein expression by 0.405±0.086 for Notch 1 and 0.414±0.909 for HES-1, whereas DAPT treatment decreased protein levels by 0.221±0.107 and 0.348±0.108 for Notch-1 and HES-1, respectively. Thus, the expression of Notch 1 and HES-1 proteins was more readily decreased in the Marimastat treated renal carcinomas than in those treated by DAPT. Notably, the same concentrations of each inhibitor were used for treatments. Further analysis revealed that Marimastat treatment more significantly decreased the two proteins than DAPT treatment (786-O Notch1 P<0.05 Hes-1 P<0.05; OS-RC-2 Notch1 P<0.05 Hes-1 P<0.05) (Table 2). These data suggest that Marimastat more effectively inhibits activation of the Notch pathway.

**Table 2:** The decrease protein level of Notch1 and Hes-1 after treatments in renal cell lines

	**Notch1 with Marimastat**	**Notch1 with DAPT**	**P value**	**Hes-1 with Marimastat**	**Hes-1 with DAPT**	**P value**
786-O cell	0.397±0.126	0.364±0.068	P<0.05	0.411±0.096	0.391±0.099	P<0.05
OS-RC-2 cell	0.405±0.086	0.221±0.107	P<0.05	0.414±0.909	0.348±0.108	P<0.05

The expression of Notch 1 and HES-1 proteins was more readily decreased in the Marimastat treated renal carcinomas than in those treated by DAPT (786-O Notch1 P<0.05 Hes-1 P<0.05; OS-RC-2 Notch1 P<0.05 Hes-1 P<0.05).

### The impact on invasion of 786-O and OS-RC-2 cells is greater with the ADAM-17 inhibitor Marimastat than the γ-secretase inhibitor DAPT

**Figure 3:**
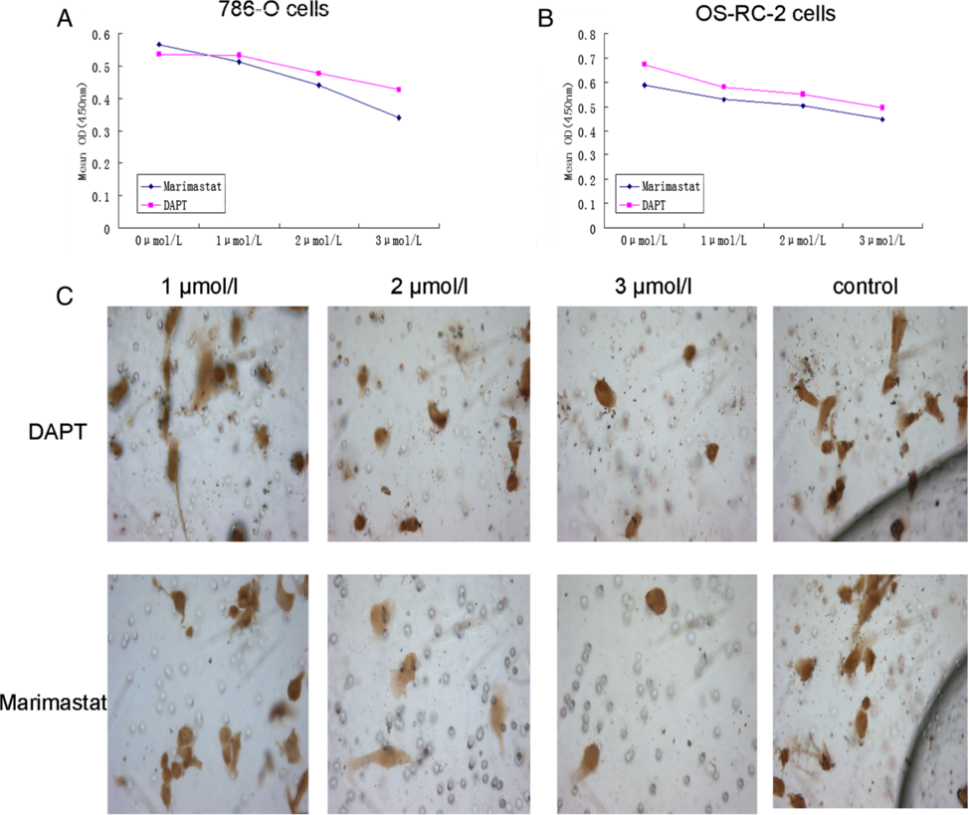
**Inhibition of either ADAM-17 or γ-secretase reduces proliferation of renal carcinoma cell lines. A**–**B**: 786-O (**A**) and OS-RC-2 (**B**) were treated with either Marimastat or DAPT at different doses then proliferation was measured by CCK-8 assay, the control group is no treatment. The mean cell activity (OD) of three experiments is presented (P<0.05). **C**: Expression of 786-O cells in the transwell assay by different doses of two types of inhibitor treatment cells.

After treatment of the two cell lines with different doses of either Marimastat or DAPT (1–3 μmol/L), we found the ODs were readily decreased in both cell lines when compared with the DMSO treated control. Moreover, we found that the mean OD value of Marimastat-treated 786-O cells was lower than that for cells treated with the same dose of DAPT (1 μmol/L = 0.529 vs 0.579; 2 μmol/L = 0.502 vs 0.549; 3 μmol/L = 0.446 vs 0.495; and control group = 0.589 vs 0.672). Similar results were obtained using OS-RC-2 cells (1 μmol/L = 0.514 vs 0.533; 2 μmol/L = 0.442 vs 0.477; 3 μmol/L = 0.340 vs 0.428; and control group = 0.566 vs 0.536). Statistical analysis revealed that the two inhibitors both significantly decreased the invasive ability (P<0.05, P<0.05) (Figure 3A and B). However, under the same dose conditions, Marimastat rendered a greater impact on the two types of renal carcinoma cell lines than did DAPT (P<0.05).

### ADAM-17 inhibitor Marimastat more effectively impairs invasion of 786-O cells than the γ-secretase inhibitor DAPT

We tested the invasive capacity of the renal carcinoma cells, 786-O, treated with either Marimastat or DAPT at concentrations of 1 μmol/L, 2 μmol/L, and 3 μmol/L, by Transwell assay. Treatment with either Marimastat or DAPT reduced the number of 786-O invasive cells in a dose-dependent manner when compared with the non-treated control group (Figure 3C). Notably, the drug-induced reduction in invasive cell number was significantly more potent with Marimastat treatment than with DAPT (Table 3) (p<0.05). Thus we demonstrated that with the same dose, the ADAM-17 inhibitor Marimastat more effectively impairs invasion of 786-O cells than the γ-Secretase inhibitor DAPT.

**Table 3:** Result of Transwell assay in 786-o cell treated by different inhibitors

	**Marimastat**	**DAPT**
Concentration		
1μmol/L	7.80±1.64	15.8±3.19
2μmol/L	3.4±0.55	10.8±1.72
3μmol/L	1.2±0.84	4.4±0.55
Control	34.2±1.50	31.8±3.19

In the Transwell assay, the number of 786-o cells penetrating Matrigel decreased with the increasing concentration of Marimastat and DAPT, whereas Marimastat had more effect under the same concentration(P<0.05), which indicates that MARIMASTAT is more capable of thwarting the invasion of 786-o cells.

### ADAM-17 inhibitor Marimastat more effectively increases the apoptosis rate in 786-O cells than the γ-secretase inhibitor DAPT

**Figure 4:**
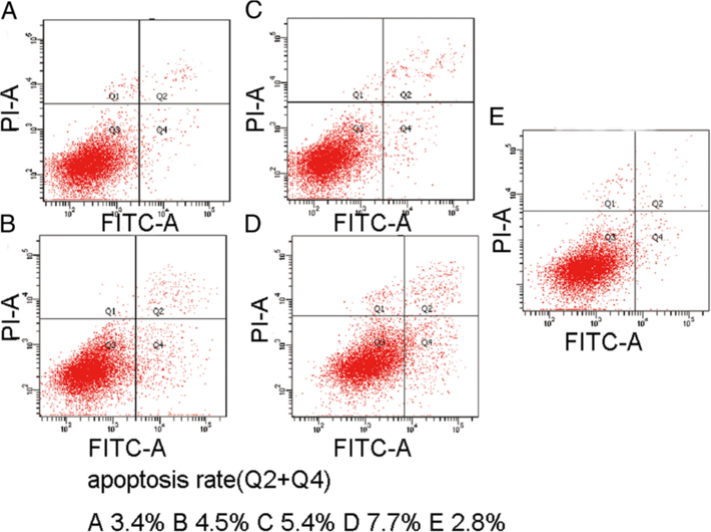
**The effect of Marimastat and DAPT on apoptosis of renal carcinoma cells. A**–**E**: Flow cytometry was performed after Annexin-PI staining to observe the 786-O apoptosis rate after treatment with either of the two inhibitors at two doses. 1 μmol/L DAPT (**A**) and Marimastat (**B**); 3 μmol/L DAPT (**C**) and Marimastat (**D**); DMSO control (**E**).

To study the effect of Marimastat and DAPT on the apoptosis of 786-O, Annexin-V-PI staining and flow cytometry were conducted after cells were treated with inhibitors (1 μmol/L and 3 μmol/L treatment), or DMSO as a control. Analysis of Annexin V-PI staining showed apoptotic rates of 3.4% and 5.4% for 786-O after DAPT treatment with 1 μmol/L and 3 μmol/L, respectively (Figure 4A and C), and 4.5% and 7.7% following Marimastat treatment with the same doses (Figure 4B and D). Lower levels of apoptosis (2.8%) were detected in the control group (Figure 4E). The following statistical analysis showed that the apoptosis rates of 786-O after Marimastat treatment was greater than that attained after treatment with DAPT at the same concentrations (P<0.05). This test demonstrated that the ADAM-17 inhibitor Marimastat exhibits higher specificity and greater impact on the apoptosis rate of 786-O than the γ-secretase inhibitor DAPT.

## Discussion

Notch signaling and its receptor play an important role in tumor occurrence and development [7-9]. Because this pathway signals for cell apoptosis and revascularization in renal carcinoma, many researchers focus on the inhibition of Notch. Sjölund’s and later researchers have shown that activation of the Notch pathway reinforces invasion of renal carcinoma [10-14]. ADAM-17 which is the key enzyme has been reported to be highly-expressed in renal carcinoma in the mRNA level in 27 patient samples [15]. However, in this study, 67 renal carcinoma tissues were examined and found to express high levels of ADAM-17 in different TNM stages, especially the advanced stages, T3 and T4. Because ADAM-17 is involved in Notch activation, this finding suggests that ADAM-17 activation of Notch correlates with RCC progression. Indeed, Aparicio’s and Buzkulak’s research demonstrated that Notch 1 protein levels increase in renal carcinoma in association with clinical staging [16,17]. These findings manifest the important role of the Notch pathway in the development of renal carcinoma. In our research, we demonstrate that high expression of ADAM-17 is closely related to the malignancy of renal cancer. Moreover, the consistent expression trend of ADAM-17 and Notch1 proteins suggest that a positive relationship exists between the two.

Marimastat is the only metalloprotease considered to be able to inhibit the ADAM-17 protein [18]. By Murthy’s research, it was demonstrated that ADAM-17 could suppress the activation of the Notch signal system [19]. Furthermore, Marimastat has been acknowledged for its impact on tumors through down-regulation of the Notch pathway by inhibiting ADAM-17. A growing number of new ADAM-17 inhibitors have also emerged in recent years including IK682 [20]. The recent research on γ-secretase inhibitors has revealed that it may also work as a Notch pathway inhibitor and be useful in treatment of malignant tumors where this pathway is deregulated [21,22]. In our research, both Marimastat and DAPT down-regulated the expression levels of Notch1 and HES-1 proteins. Indeed, our data demonstrates that these two drugs inhibit the Notch pathway in a dose-dependent fashion (Figure 1C and D). Importantly, we found that Marimastat more effectively blocked the Notch pathway, when compared with the effects of DAPT at the same dose. This suggests that in RCC cell lines, blocking ADAM-17 can decrease expression of the Notch pathway and its downstream target genes, more efficiently than γ-secretase inhibition.

The Notch pathway has been published to induce tumor proliferation and increase invasiveness. For example, Wu reported that in breast cancer, the Notch pathway can induce the proliferation and invasion [23,24]. We used Marimastat and DAPT for the targeted inhibition of ADAM-17 and γ-secretase, respectively. We observed that proliferation of 786-O and OS-RC-2 RCC cells was significant decreased after treatment with either inhibitor, especially after use of greater concentrations. This suggests that in RCC cell lines, inhibition of the Notch pathway can reduce the proliferative ability. Importantly, when treatment effects of Marimastat and DAPT, used at the same concentrations, were compared, Marimastat was found to more significantly decrease proliferation than DAPT. This trend also appeared in the transwell invasion assay performed using 786-O cells, where the number of cells able to pass through the polycarbonate membrane was more significantly impaired with Marimastat than DAPT at the same concentration (Figure 3C). Thus, our data confirms that in RCC, inhibiting the Notch pathway can cause inhibition of cell proliferation and decrease invasive capacity. For the first time, we demonstrated that the effect of ADAM-17 inhibition is better than that achieve by inhibition of γ-secretase in RCC cell lines. In our flow cytometry assay, it was clearly found that inhibition of the Notch pathway through the two types of inhibitors caused increased apoptosis (Figure 4), where again the effect of Marimastat was more pronounced than that of DAPT. Thus, our data suggest that inhibition of the Notch signaling pathway can impair both proliferation and cell invasion ability, and increase the apoptosis rate of RCC. These effects were best when ADAM-17 was suppressed using Marimastat than if the γ-secretase inhibitor DAPT was used, suggesting that Marimastat is a highly potent inhibitor of the Notch pathway.

In our research, we reveal that blocking the expression of ADAM-17, which is needed for activation of Notch via cleavage of the S2 site, is more specific and effective than inhibition of γ-secretase-mediated cleavage of the S3 site in RCC. We believe that the reason for this is that as ADAM-17 is not a transmembrane protein, activation of ADAM-17 could lead to the stimulation of a variety of intracellular pathways including the Notch pathway and its activators, such as G-protein coupled receptors (GPCR) and PKC [25]. Thus inhibition of ADAM-17 may suppress other intracellular pathways which can affect the Notch pathway such as EGFR [26]. Another reason why Marimastat exhibited superior ability to decrease the malignant phenotype, could be because the S3 sites in Notch that are cut by γ-secretase are located in the transmembrane region, and are therefore only activated downstream of the Notch pathway. Therefore, inhibition of ADAM-17 can relay a better and more specific effect, and the ADAM-17 inhibitor Marimastat appears to be a better targeted inhibitor. We expect that the results of this study can provide a new way for a future targeted therapy treatment against RCC especially through inhibition of the Notch signal system.

## Competing interest

The authors declare that they have no competing interest.

## Authors’ contribution

ZG carried out the molecular genetic studies, participated in the sequence alignment and drafted the manuscript. ZG and HJ carried out the experimental assay. XJ participated in the design of the study and performed the statistical analysis. ZG and XJ conceived of the study, and participated in its design and coordination and helped to draft the manuscript. All authors read and approved the final manuscript.
